# “Climate-friendly” diets from an allergy point of view 

**DOI:** 10.5414/ALX02471E

**Published:** 2024-05-03

**Authors:** Imke Reese

**Affiliations:** Private Practice for Dietary Advice and Nutrition Therapy with Special Interest in Adverse Reactions to Food, Munich, Germany

**Keywords:** calcium, iondine, UPF (ultra-processed foods), pollen food syndrome

## Abstract

Since the EAT-Lancet Commission’s call for a change in diet towards more plant-based foods, especially protein sources, this so called “Planetary Health Diet (PHD)” has been widely discussed. While for some the reduction in animal foods is not enough and vegan diets are advocated to save the climate, others are sounding the alarm that the reduction is too drastic and that the PHD makes it impossible to provide a diet that meets our needs (of essential nutrients). In addition to climate aspects, health benefits often cited to justify the PHD do not take into account that vegetarians/vegans differ from the general population by far more factors than the reduction or elimination of animal foods. Also not sufficiently discussed is the fact that a diet which excludes or severely restricts animal foods is also associated with health risks if critical nutrients are not adequately covered. Moreover, the challenge of meeting protein requirements is underestimated by many. The food industry has responded to the trend towards more plant-based foods by massively expanding the range of highly processed or ultra-processed vegan foods. These – vegan or not vegan – are suspected of being partly responsible for the development of non-communicable diseases. In addition to general criticism regarding the usefulness of advertising the PHD, the replacement of animal protein sources with plant-based sources notably harbors a number of additional relevant risks for allergy sufferers so that the latter should be classified as an unfavorable target group for the implementation of the PHD recommendations.

## What is the basis for recommendations regarding a climate-friendly diet? 

Over a period of 3 years, the EAT-Lancet Commission, encompassing experts from 16 countries, developed strategies for changing the global food system in order to feed the world’s population in 2050 and to meet the UN Sustainable Development Goals (SDGs). These strategies were published prominently in the Lancet [1] and in a summary report for the general public [2] in 2019 and have been the subject of intense debate ever since. 

In order to meet the enormous challenge, two goals were defined: (1) healthy diets and (2) sustainable food production.

1. The proposals for a “Planetary Health Diet” (PHD) are intended to change the diet of the entire world‘s population by 2050 towards a largely plant-based diet. The recommendations for an average person with an intake of 2,500 kcal are illustrated by a plate model and a table with possible ranges and recommended average quantities for each food group. While the recommended intake of whole grains is fixed without a range, animal foods typically range from zero (no consumption) to a low maximum intake.
2. Ideally, production practices should include “a 30% increase in nitrogen use efficiency, and 50% recycling rate of phosphorus: a phase-out of first-generation biofuels, and implementation of all available bottom-up options for mitigating food-related greenhouse gas (GHG) emissions. For biodiversity, it was assumed that land use is optimized across regions such that it minimizes impacts on biodiversity.” [2]

According to the authors, the goals set can be achieved, but this would require a significant change towards plant-based dietary patterns, a significant reduction in food loss and waste, and significant improvements in the area of food production. However, according to the various scenarios presented in the “Summary report”, the effects of the three measures on the projected consequences for 2050 in terms of greenhouse gas emissions, land and water use, nitrogen and phosphorus use, and loss of diversity, the desired changes can be achieved primarily through optimization in the area of food production [2]. Surprisingly, the change in dietary patterns alone would only have a positive effect on emissions, but no relevant effect on other goals and even a negative effect on biodiversity. Nevertheless, PHD is considered by many to be the benchmark for a climate- and environment-friendly diet which additionally is favorable for their own health. 

## How sustainable is the implementation of the PHD recommendations? 

As animal husbandry is considered to be the main cause of climate-damaging consequences in the area of nutrition, the PHD aims to replace a large proportion of animal-based foods with plant-based foods. The assumed health benefits of reduced meat consumption claimed will be discussed in the next section. 

While the scenarios of the EAT-Lancet Commission identify a change in diet as a key factor in reducing greenhouse gas emissions, current FAO calculations show that the potential for reducing emissions of CO_2_, NO_2_, and CH_4_ does not lie primarily in changing diets but above all in increasing productivity, breeding, and improving animal health [3]. The reduction of food waste is also rated as more relevant than a change in diet. Carbon sequestration, which takes into account the fact that grassland binds CO_2_, ranks fourth in terms of potential savings. The climatic conditions in Europe are well suited to ruminant husbandry. According to a report published by the German Federal Environment Agency on “Perspectives for environmentally friendly livestock farming” in Germany [4], a pure grassland diet is possible. It preserves biodiversity and “hardly competes with humans for food”. 

Looking at “water consumption and water scarcity”, WWF calculations show that 82% of the current total water consumption due to artificial irrigation in Germany is attributable to the production of plant-based foods and 18% to animal-based foods [5]. As the majority of plant-based foods are imported (63% of vegetables, 80% of fruit and pulses) and therefore often come from regions with water scarcity, 96% of the “German water scarcity footprint” is due to plant-based foods and only 4% to animal-based foods. 

In a country like Germany, diets can be improved regarding sustainability by taking into account regionality and seasonality rather than by switching to a diet that attempts to cover protein requirements primarily through protein sources that do not find optimal growing conditions in Germany. 

Following a wide range of criticism and worldwide consultations with various stakeholders, the recently updated EAT-Lancet 2.0 Global Consultations now include numerous aspects such as region-specific dietary recommendations, promotion of local and seasonal food production, promotion of regenerative farming practices, efficient resource use and waste reduction, but also cultural preservation and embracement of indigenous knowledge [6]. 

## Is the Planetary Health Diet beneficial to health? 

Regarding the influence on health, opinions differ widely on the assessment of plant-based diets: for some, the considerable reduction in animal foods proposed as part of the PHD leads to the perception that a diet without any animal components is even more beneficial, while others sound the alarm that the reduction is too drastic and that (micro-)nutrient adequacy is not possible with the PHD [7, 8, 9]. 

### Claimed health benefits of vegetarian and vegan diets 

The authors and proponents of the PHD claim health benefits of meat-free diets, particularly with regard to cardiovascular disease [10, 11]. On the other hand, recent meta-analyses using GRADE have concluded that these claims regarding meat consumption are based on low to very low evidence and it is not clear if and to what extend benefits can be expected [12, 13, 14]. Moreover, the FAO concludes, based on “evidence from the evolutionary past of Homo sapiens”, that “higher dietary intake of terrestrial animal source food were associated with increased stature, brain size and longevity”, and that consumption “within appropriate dietary patterns can make important contributions to reducing stunting and wasting in children under five years of age, low birth weight, anemia in women of reproductive age (15 – 49 years), overweight in children under five, and obesity and diet-related non-communicable diseases (NCDs) in adults” [15]. 

Proponents of meat-free diets often ignore evidence that observed health benefits can be largely explained by accompanying lifestyle factors (increased exercise, non-smoking, health consciousness, etc.) and a more selected diet, but not by abstaining from meat [10, 16]. Accordingly, Mediterranean diets, which do not eliminate anything per se but are characterized by a high variety of freshly prepared food, perform better in meta-analyses with regard to health outcomes than vegetarian or even vegan diets. Not only the risk of cardiovascular disease, but also of mortality in general, cancer, neurodegenerative diseases, and diabetes can be reduced with a Mediterranean diet with convincing or highly suggestive evidence [17]. 

Looking at industrialized countries such as Germany, the cause of lifestyle diseases, above all overweight and obesity, is largely due to a sedentary lifestyle, overeating and malnutrition, lack of sleep, and lack of sunlight. Insulin resistance as a result of these unfavorable lifestyle factors is a key factor of most lifestyle diseases [18]. It is doubtful whether a diet such as the PHD providing 51% of energy from carbohydrates and only 14% from protein sources, which are predominantly plant-based and therefore energy-dense, can effectively counteract the increasing insulin resistance in this population. 

### Focus on critical nutrients in plant-based diets 

A diet which excludes animal foods cannot meet requirements without supplements and is therefore associated with high risks to health if critical nutrients are not adequately covered. This fact and the difficulty of meeting protein requirements, which is often underestimated, have already been discussed in detail in the position paper of the DGAKI Food Allergy Working Group “Vegan diets from an allergy point of view” [19]. However, people who do not completely exclude animal foods but consume them only in small amounts, are also at great risk of not meeting their needs. This group of people often is not aware of the need to select their food carefully and that they may need to supplement some critical nutrients despite occasionally consuming animal foods. On the other hand, it is undisputed that also people who do not avoid anything (omnivores) not automatically meet their needs [20, 21, 22], even if micronutrient-rich foods come predominantly from animal sources [23]. Ultimately, conscious selection, composition, and preparation of food is crucial in all diets. 

Comparisons between different dietary patterns clearly show that protein intake decreases on average when less animal-based foods are eaten [20, 21]. This observation is all the more alarming as the protein requirement increases the more plant-based the diet becomes (see also [19]). A (too) low protein intake often results from replacing milk and dairy products with plant-based “alternatives”, which not only reduces the protein quality of the diet (except for soy drinks), but also the intake of calcium and iodine [24]. The more milk and dairy products are replaced by plant-based “milk alternatives”, the more critical calcium intake becomes [21, 25], even if the drink is calcium-fortified, as the added calcium from plant-based drinks is often insufficiently absorbed [26]. The only exception in this respect are calcium-fortified soy products [27, 28, 29]. The iodine supply is also at risk on the basis of the PHD [21, 30, 31]. As long as milk is not replaced by plant-based drinks, 85% of the adult recommendations can be provided with the PHD, but only 51 – 64% of those in pregnancy [30]. If animal-based foods are completely avoided, an adequate iodine supply is only possible via supplements [30]. 

### Increase in plant-based ultra-processed foods as a risk factor 

With the ambition to transform the global food system from animal to plant-based protein sources, the development of ultra-processed plant-based foods has increased dramatically [19, 32]. Ultra-processed foods (UPF) are often characterized by high levels of starch and sugar, refined fat, salt, and a lack of nutrients. By definition, the reason for their production also differs: they aim to be “convenient” (durable and ready to consume), tasteful (with the aim of eating a lot of it), and profitable (cheap ingredients, but added value when sold) [33, 34]. Even if manufacturers of plant-based UPFs may have a genuine environmental concern, there is some evidence that profit motives are not insignificant in the development and production of UPFs [32, 35]. 

A comparison of traditional diets (flexitarian, vegetarian, vegan) with dietary patterns using highly processed substitutes instead of meat, eggs, dairy products, and also traditional preparations of legumes and nuts, showed a significant increase in saturated fat and salt, a slight increase in sugar, a reduction in fiber (the difference was particularly pronounced in vegan diets), vitamin B12, calcium, potassium, magnesium, and zinc [36]. With the increased consumption of UPFs, all three diets change towards a typical “Western diet”, which is associated with a high risk of NCDs, and lose the advantages (lots of fresh and freshly prepared vegetables and fruits, nuts, legumes, etc.) they are originally associated with. 

To underline the fact that the quality of the diet is not defined by what is eliminated, but by careful food selection and preparation, a distinction is now often made between “healthy vegetarian diets” and “unhealthy vegetarian diets” [37]. Prescott et al. [32] warn that many of these products can potentially compromise health at all scales of people, places, and planet, and that more attention needs to be paid to the impact of these products on human and environmental health. This aspect was also addressed in the global consultations with various stakeholders and included in the EAT-Lancet 2.0 Global Consultations [6]. 

## Planetary Health Diet from an allergological perspective 

The replacement of animal food or protein sources with plant-based sources entails a number of risks for people affected by allergies, which have already been presented and discussed in detail in the position paper of the DGAKI Food Allergy Working Group “Vegan diets from an allergy point of view” [19] and will therefore only be outlined here. 

With increasing age, food allergies are increasingly elicited by plant foods: in fact, wheat, legumes, nuts, and celery are the leading anaphylaxis triggers in adulthood alongside shellfish [38]. The fact that only some legumes (soy and lupine) and seeds (sesame) are subject to mandatory labeling and therefore need to be highlighted in the ingredient list, is particularly problematic [19, 39]. In particular peas, which have been increasingly used as a source of protein in recent years due to their diverse technological properties are not subject to mandatory labeling. Recently, a Bet v 1 homolog was described also for pea [37]. This may, similar to Gly m 4 in soy, cause anaphylactic reactions, when consumed in plant-based drinks [19, 40]. 

The results of a French working group that investigated the clinical relevance of sensitization to legumes in peanut-allergic children should be noted: 28% (n = 34) of the sensitized children were found to be allergic to one or more legumes [41]. The main triggers were lentil (n = 8), lupin (n = 12) and pea (n = 8), fenugreek seeds (n = 6), soy (n = 5), and chickpea (n = 2). Many of these food allergies were already known. In contrast, the outcome of the oral food challenges with lupin was unexpected: 10 out of 18 challenged children were allergic, although the majority reported that they had not yet eaten lupin or were unable to say. Also, the comparatively high number of allergies to fenugreek seeds, an allergen that has often been underestimated to date, must also be taken into account, especially as this – like lentil, pea, and chickpea – is not subject to mandatory labeling. Due to the lack of highlighting these legumes, ingredient lists need to be read much more carefully. It is also possible that relevant allergens are “masked” behind the term plant protein. 

Patients with wheat allergy, especially those with cofactor-triggered wheat anaphylaxis, should also be aware of the risk that wheat can be more frequently found in a plant-based diet, not only as a grain but also as a source of protein, e.g., in the form of seitan. 

Even people with an often (wrongly) trivialized birch pollen-associated food allergy are likely to find it more difficult to switch to PHD than those without food allergies. Especially if “climate-friendly” is understood to prefer regional fruit and vegetables, this is likely to reduce intake to cooked or sufficiently heated forms of many fruits, vegetables, and nuts. In the case of LTP (lipid transfer protein) allergies, cooking would not be an option either, as heating is not sufficient to destroy the relevant allergens. As soy drinks and soy yoghurt cannot be safely consumed by birch pollen allergic patients, it is extremely challenging to meet protein and calcium requirements in this group of patients. 

## Conclusion 

Based on the above, it is clear that allergy sufferers are an unfavorable target group for implementing the PHD recommendations. However, this does not mean that a climate-conscious diet or even a climate-conscious and health-oriented lifestyle is not possible for people with food allergies. A conscious lifestyle and the optimization of agricultural production practices with regard to sustainability are obviously much more important than the implementation of PHD. Resources such as energy and water can also be used more sparingly or more consciously in everyday life, waste can be avoided, consumer behavior can be reconsidered, and sustainable agriculture, including animal husbandry, can be supported. 

## Funding 

No funding. 

## Conflict of interest 

I. Reese declares the following conflicts of interest outside the submitted work: Honoraria: AGPAS e. V., Aimmune Therapeutics Germany GmbH, AlbertZwei media GmbH, ALK-Abello Arzneimittel GmbH, BVDD e. V., DAAB e.V., DAEM e.V., Danone Deutschland GmbH, DDG e.V., DGAKI, GPA e.V., GEKA mbH, GWT-TUD GmbH, HAL Allergie GmbH, Helmholtz-Zentrum München, InfectoPharm Arzneimittel und Consilium GmbH, KneippÄrztebund e.V., Medical Project Design, MVS Medizinverlage, Nestlé Deutschland AG, Norsan GmbH, Novartis Pharma GmbH, RG Ärztefortbildung, Sanofi-Aventis Deutschland GmbH, Uniklinikum Augsburg, Uniklinikum TU München, Uniklinikum Marburg, VDD e.V., VDOe e.V., Vfed e.V. 

Publishers: Dustri, de Gruyter, Springer, Thieme, Münchner Verlagsgruppe. 

Reimbursement of travel costs: DGAKI, EAACI, GA2LEN GAFA. 

Advisory board activity: DAAB, DGAKI. 
